# Infants’ Understanding of Object-Directed Action: An Interdisciplinary Synthesis

**DOI:** 10.3389/fpsyg.2016.00111

**Published:** 2016-02-09

**Authors:** Scott J. Robson, Valerie A. Kuhlmeier

**Affiliations:** ^1^Centre for Neuroscience Studies, Queen’s UniversityKingston, ON, Canada; ^2^Department of Psychology, Queen’s UniversityKingston, ON, Canada

**Keywords:** infant goal attribution, mirror neurons, object cognition, social cognition, action prediction

## Abstract

Recognizing that the object-directed actions of others are governed by goals and intentions is a crucial component of human interaction. These actions often occur rapidly and without explanation, yet we learn from and predict the actions of others with remarkable speed and accuracy, even during the first year of life. This review paper will serve as a bridge between several disparate literatures that, we suggest, can each contribute to our understanding of how infants interpret action. Specifically, we provide a review not just of research on infant goal attribution *per se*, but also incorporate findings from studies on the mirror neuron system and infant object cognition. The integration of these various research approaches allows for a novel construal of the extents and limits of early goal attribution – one in which the importance of the entire action context is considered – and points to specific future research directions.

## Introduction

For 20 years, we have known that infants are able to encode the object-directed actions of others in terms of their goals (e.g., Gergely et al., 1995; Meltzoff, 1995; Woodward, 1998). In the intervening years, a great deal of thought and experimental effort has gone into untangling exactly how it is that infants produce these goal attributions. Indeed, the ability to attribute goals to others – a component of social learning, prosocial behavior, and communication, with consequences throughout the lifespan – has been of interest to researchers in other fields as well who, in turn, bring their own theoretical backgrounds and techniques. The cross-disciplinary interest is in part due to the complexity of seemingly simple actions. For example, consider an infant who is witnessing an adult reach toward an apple. In addition to gathering evidence that the infant construes the action as being goal-directed, researchers might be interested in how that action is represented at a cellular level in the infant’s brain, how the infant garners information from the shape of the experimenter’s hand, or how the features of the apple are represented and maintained in the infant’s memory. These are all interesting and valuable approaches to our understanding of object-directed actions, but there have been limited attempts to synthesize the contributions of different fields.

At best, a fragmented view of the research findings is limiting: to the observing infant, the topics of these separate lines of research all represent viable streams of complementary information. At worst, this fragmentation can lead to poorly controlled experiments as researchers may not be well versed in the theoretical and methodological insights from other related areas. Indeed, as these approaches all represent rich and active fields of study, maintaining a current understanding of these issues is a daunting task. However, infants obtain and implement their ability to represent the goals of others in a world that is complex and uncontrolled, and so piecing together how these streams of information interact together is crucial to forming a true understanding of infant goal attribution.

**FIGURE 1:**
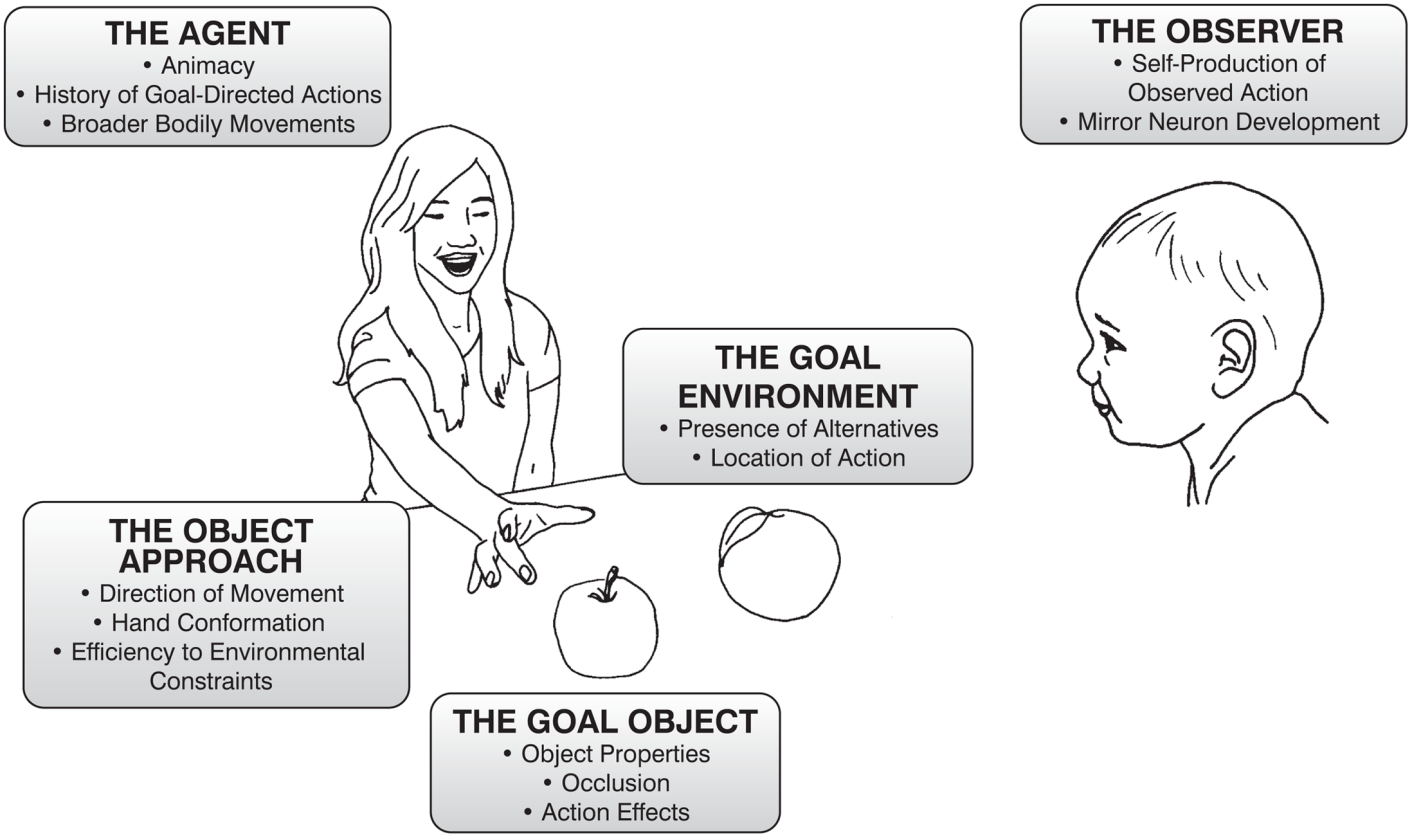
**Description of the elements of an object-directed reach that can influence infants encoding of the goal of that reach**. The following paper will discuss each of the categories listed above and expand upon how the listed factors influence infant’s judgements.

The aim of the following review is to synthesize the work from the last 20 years (approximately) that is explicitly related to infant goal attribution with research from the neuroscientific study of human and non-human animals and object cognition. To do so, we have organized the review into five broad (and subdivided) categories of influence that together constitute the *action context*: the experience and brain maturation of the infant observing the goal-directed action, the agent who is enacting the goal-directed action, the components of the action taken to achieve the goal, the nature of the goal-object itself, and the environment in which the goal-directed action occurs (**Figure 1**).

Before starting, however, it is important to present a few caveats. By ‘goal attribution,’ we specifically refer to a process by which an infant forms either an expectation or a prediction regarding the target and nature of another’s ongoing or future action. Though ‘goal attribution’ in this sense could be applied to a number of types of events, in the following paper it refers almost exclusively to an object-directed^1^ reaching action. Second, categorizing aspects of the action context is not meant to imply orthogonality between them. As will be clear throughout the review, there is substantial overlap between these categories. Finally, we are not proposing that that this organization reflects any sort of functional model of infant goal attribution, nor do we suggest that the categories of influence on infant goal attribution are necessarily processed simultaneously or even in every circumstance. Rather, we have synthesized a wide variety of studies on a wide range of influences on infants’ attribution of goals and present them without attempting to make strong claims as to the relative importance of each type of influence. In part, this is due to a lack of the empirical evidence necessary to do so. However, the primary goal in using this organizational framework is to show the potential sources of information vying for infants’ attention in a way that draws attention to the importance of the entire context in which goal-directed action occurs.

## The Observer

In the action context discussed in this paper, the observer is an infant who is watching an agent perform a goal-directed action. In a seminal study, Woodward (1998) demonstrated that infants at 9-months of age are sensitive to the goals of others. In a procedure that has since become widely utilized, 6- and 9-month-old infants first watched as a hand moved out onto a table containing two objects. The hand would approach and grasp one object from the pair, and would remain in this position until the end of the trial. This action was repeated upon the same object until the infants habituated (i.e., their looking time to the event decreased to a predetermined criterion). At this point, the locations of the objects were switched, and the hand reached either toward the same object in a new location or the previously untouched object in the previously reached for location. Infants dishabituated (increased looking behavior) to the latter, indicating that they encoded the original reach as directed to a particular object. It has subsequently been shown that infants make predictions, as measured by eye gaze, regarding which object will be reached for within this paradigm (Cannon and Woodward, 2012).

Woodward’s experimental procedure has been remarkably fruitful and has revealed much about how infants view the actions of others. For instance, some researchers, performing variations on Woodward’s (1998) classic experiment have shown that even younger infants attribute goals to others (e.g., 5 months, Luo and Baillargeon, 2005). Tests with younger infants have found more mixed results (Sommerville et al., 2005; Luo, 2011). This change in behavior over time, which appears to center on about 5 months of age, suggests a likely role for both experience and brain maturation. In this way, age is one characteristic of the observer that influences the way they perceive the actions of others.

### Self-Production of the Observed Action

Several studies from Sommerville et al. (2005) have emphasized the role of action experience in the development of goal attribution. Sommerville et al. (2005) placed Velcro mittens on the hands of 3-month-old infants and gave them time to interact with Velcro objects in their vicinity. Typically, infants begin to reach for objects in their vicinity sometime between 3- and 5-months of age, so the infants in this study were old enough to extend their hands and contact the objects, but not yet coordinated enough to have much first-hand experience with reaching and grasping prior to their session with the ‘sticky mittens’ (von Hofsten, 1991; Thelen et al., 1993; Berthier and Keen, 2006). When these infants took part in a version of Woodward’s (1998) experiment featuring an experimenter wearing the same gloves, they expected the actor to act consistently following the switch in target object locations, while inexperienced infants showed no expectations. In particular, the role of first-person experience seems to be particularly important, as infants who have simply observed others using the Velcro mittens show no expectation for consistent goal-related action (Woodward, 2009).

Further, there is a relation between the ability to produce an action and to predict the goal of an action (Falck-Ytter et al., 2006). In this study, infants watched a video in which an experimenter reached toward objects on a stage and lifted them across the stage and into a container. Adults and 12-month-olds both showed predictive gaze toward the target. Six-month-olds, however, lacking the ability to perform such an action themselves, did not exhibit anticipatory gaze toward the target. Kanakogi and Itakura (2011) similarly demonstrated a relation between object-directed action prediction and the ability to produce object-directed reaches by examining infant eye movements. Four-month-old infants, who lacked the ability to perform grasping actions, did not make anticipatory gazes, but 6-, 8-, and 10- month-old infants, did gaze toward the target of the reach before the arrival of the experimenter’s hand.

More recently, infants’ ability to predict the goal of specific types of object-directed reaching actions were measured alongside their ability to perform the grasping actions themselves (Ambrosini et al., 2013). Six-, 8-, and 10-month-old infants watched a video in which a small ball and a large ball were present on a table. The infants watched as an experimenter made reaches toward the objects with either a closed fist, a hand shaped for a whole-hand grasp, or a hand shaped for a precision pincer grip. Infants are able to make whole-hand grasps at all three of these ages, but the pincer grasp typically develops around 8-months of age or later. While the experimenter made reaches to both the large and small balls with the closed fist action, the other reaches went to the appropriate target for their reach type (whole hand to large ball, pincer to small ball). All infants showed an advantage in goal anticipation toward the whole hand grasp compared to the fist-to-large ball reach. However, predictive advantage for precision grasping was only present and 8- and 10-month old infants, and was greater in the 10-month olds, consistent their increased experience with this action over the younger infants. Relatedly, 10-month-old infants who received direct experience with using a cane to reach a toy were later sensitive to the goal of a cane-using actor, yet infants who had observed another person training, or who had received no training, were not (Sommerville et al., 2008).

In addition to experience, the infant’s own engagement also seems to play a role in their growing understanding of others actions. Infants are not passive observers of other’s actions, though often positioned that way in experimental settings, and some researchers have made a case that infant participatory role in others actions is more crucial than it is typically given credit for (Reddy and Uithol, 2015). Moll et al. (2007) demonstrated that 14-month-old infants had stronger recognition of the goal of an agent if they had engaged directly with an experimenter as opposed to watching that experimenter have an interaction with another person. Others have shown that mother-infant interaction style can have an influence on the age at which infants are able to demonstrate goal attribution (Hohenberger et al., 2012; Licata et al., 2014). The infant’s own part in influencing the goal directed actions of others is clearest when considering that in the early months of life (and to a lesser extent into the toddler years) the goal-directed actions most frequently observed by an infant will feature the infant themselves as the target of that action (picking up, feeding, diaper change, dressing, etc.). Indeed, infants make postural adjustments in anticipation of infant-as-object action from their mother as early as 2-months of age (Reddy et al., 2013). A stronger understanding of how infants level of engagement with others interacts with their own ability to produce actions will likely prove crucial to a complete picture of how goal attribution influences infants’ behavior in their day-to-day lives.

Though infants’ own production of action does seem to be important to their interpretation of others’ behavior, its precise role remains unclear. Infants also attribute goals to non-human agents (Shimizu and Johnson, 2004; Luo and Baillargeon, 2005; Johnson et al., 2007), to novel tool-use actions (Hernik and Csibra, 2015), and to actions that are biomechanically impossible (Southgate et al., 2008), situations with which infants could not possibly have first person action experience. In turn, as will be seen in section “Mirror Neuron Development,” the attribution of goals to animated, non-human agents has also called into question the dominant mirror neuron theory of action comprehension (e.g., Hamilton and Ramsey, 2013).

### Mirror Neuron Development

Another aspect of the observer that influences their perception of others’ actions is their brain development. Imaging studies of the infant brain are notoriously difficult, though it has become more and more common in recent years. A good deal of the work relating brain measures to infants perception of action has been focused on the topic of mirror neurons and action mirroring, so we will provide here an overview of this topic before moving on to examples more specific to the topic of this review.

Mirror neurons are visuomotor neurons that discharge both when an individual performs a particular action and when that individual observes another performing a similar action (Rizzolatti and Craighero, 2004). Since their discovery, mirror neurons have been posited as a mechanism by which we might understand the motor activities of others (e.g., Gallese et al., 1996; Rizzolatti et al., 1996). These neurons were first discovered in the ventral premotor cortex, area F5, of macaque monkeys (*Macaca nemestrina*) and were demonstrated to be active both during the production of an action and while witnessing another performing that action (di Pellegrino et al., 1992). The first evidence that this class of neuron exists in humans emerged almost 20 years ago, but their existence remained controversial until recently, when the mirror activity was directly observed in neurons in the brains of surgical patients (Fadiga et al., 1995; Mukamel et al., 2010). More commonly, mirror activity is observed via electroencephalography (EEG) in the desynchronization of the mu rhythm, which occurs during the production and observation of action and which has been observed in both infants and adults (Cochin et al., 1998, 1999; Rizzolatti and Craighero, 2004; Nyström, 2008). A recent meta-analysis of fMRI studies with human participants found persistent evidence for ‘classical’ mirror neuron activity in inferior frontal gyrus, ventral premotor cortex, and IPL, as well as in less expected areas such as primary visual cortex and cerebellum (Molenberghs et al., 2012). Mukamel et al. (2010) observed mirroring in SMA, as well as in more unusual areas, such as hippocampus, entorhinal cortex, and parahippocampal gyrus, which has led to the suggestion that mirror neurons may represent a widely distributed minority of neurons (Keysers and Gazzola, 2010).

Since the activity of mirror neurons was first observed, they have been posited as a potential mechanism for the understanding of action. The *direct matching hypothesis* posits that observed actions activate a resonance mechanism that directly maps the observed action onto one’s own internal motor representation of that motor action, and thus mirror neurons allow us to understand others’ actions through a simulation of their acts (Rizzolatti et al., 1996; Iacoboni et al., 1999). In one study with adults, it was shown that Transcranial Magnetic Stimulation of the hand motor area, but not of the leg area, produced deficits in predictive saccades while watching a reaching hand (Elsner et al., 2013). It has been proposed by proponents of this theory that mirror neurons represent an evolutionarily selected innate endowment (Rizzolatti et al., 1996; Gallese et al., 2007); indeed, some studies have provided support for this through the use of EEG with rhesus macaque neonates during observation and production of facial movements (Ferrari et al., 2012).

This direct matching hypothesis has been criticized recently, for a variety of reasons. For example, mimed actions (a grasp without a target) do not activate mirror neurons, yet if mirror neurons were simulating acts to determine their goal, then the mirror neurons would have to activate in order to determine that there was no goal to a mimed act (Csibra, 2005). Similarly, a number of studies have shown mirror neuron activation in response to the actions of members of a different species (Buccino et al., 2004), computer animated agents (Hamilton and Ramsey, 2013), or by a robotic claw (Gazzola et al., 2007). Additionally, studies of people with apraxia have shown a dissociation between the ability to produce and recognize actions (Negri et al., 2007; Hickok, 2009).

More recent theories regarding the role of mirror neurons in goal understanding have made attempts to incorporate the response to actions that could not be represented in the motor system. One suggestion is that there is a secondary mechanism to motor mirroring, a ‘mentalizing network,’ that attempts to represent the actions of others in terms of their underlying intentions (Rizzolatti and Sinigaglia, 2010). Under this formulation, the mirror system recognizes a reach to a cup as serving the goal of drinking water, while the mentalizing system could represent the reasoning underlying that action (e.g., to alleviate thirst or to rinse a bad taste from one’s mouth). Others have suggested that there are neurons within the motor system that are activated in response to the *goals* of produced and perceived motor acts (Gazzola et al., 2007). By this account, observers are directly matching the goals of others to their own goal representations, rather than matching the kinematics of an action to one’s own representation of that action. Support for this interpretation includes the finding that monkey mirror neurons in F5 will discharge to the closing of a set of pliers on a goal, even pliers requiring different hand movements to operate (Umiltà et al., 2008). Additionally, there may be experiential effects on mirror neurons, as these effects can only be observed after the monkeys have had extensive experience with the tools (Ferrari et al., 2005; Umiltà et al., 2008; Rochat et al., 2010; Cook, 2012). Indeed, there is evidence from human infants that suggests that first-person experience with observed actions influences motor cortex activation in response to observed actions in a way not observed following strictly observational experience with an action (van Elk et al., 2008; Gerson et al., 2015; Cannon et al., 2016).

These theories describe mirror neurons as an innate evolutionary endowment, the development or dysfunction of which during infancy has been suggested to be related to a number of phenomena beyond action understanding, including neonatal imitation (Rizzolatti et al., 2001), autism spectrum disorder (Williams et al., 2001), and language development (Rizzolatti and Arbib, 1998). However, it has also been suggested by Heyes (2010) that mirror neurons are not innate, but are instead tuned through the correlated sensorimotor experiences of observing and executing the same actions. This *associative hypothesis* reduces the role of mirror neurons in action understanding compared to the direct matching hypothesis, supposing rather that mirror neurons make up one component of many that are used to a variety of social-cognitive functions. It is suggested that mirror neurons do not ‘do’ any specific thing, but that their function is determined on an individual level based on the sensorimotor experience of that individual (Heyes, 2013). Cook (2012) proposes that associative processes are more compatible with the activation of mirror neurons in response to learned acts, such as tool use, dance, and in association with sensory stimuli. However, while the associative hypothesis does provide room for developmental and learning processes, it also does not provide a specific account of what it is that this population of neurons contributes to the production or understanding of actions.

From the research with infants that does exist to date, a model is proposed in which goals are initially identified outside the motor system through the presence in the action of various cues to goal-direction, but once they have been identified, the motor system is recruited for predictive motor simulation (Southgate, 2013). This simulation does not need to precisely match the observed action, but rather is an emulative simulation of how the goal might be achieved. Southgate supports this view with evidence that infants show motor activation to actions for which the infants could not have a corresponding motor representation (Southgate and Begus, 2013). In this sense, experience matters in that it provides a template for the prediction, but the experience does not necessarily have to match the observed action.

### Summary and Outstanding Questions

There is evidence that infants’ own experience with the production of object-directed actions has an influence on their ability to understand and predict the ongoing actions of another. However, the attribution of goals in situations in which infants could not possibly have had experience suggests that action production is not entirely required for goal attribution, or that some experiences can be extrapolated into an understanding of otherwise seemingly impossible situations. There must also be a mechanism for the programming or reprogramming of mirror neurons through motor experience, and there is some evidence to suggest that this may relate to first-hand experience rather than observation (Gerson et al., 2015; Cannon et al., 2016). The evidence for the importance of experience is compatible both with the converging data suggesting that action mirroring in the motor cortex is in some way involved in goal attribution and the more recent suggestion that mirror neurons may be sensitive to goals rather than to particular actions.

Given the lack of concrete evidence for the existence of this population of neurons in the brains of human infants, it is unclear whether researchers are observing true mirroring (neurons firing in response to the same action, both when produced and observed) simply motor activation in response to object-directed actions (some neurons firing in relation to produced actions, adjacent neurons responding to observed actions). However, providing a definitive answer to this question will require great technological advancements to achieve the required spatial resolution in a non-invasive fashion. Here, our aim is not to argue against the existence of mirror neurons in human infants, but merely to call for caution in the interpretation of less direct measures of neural activity, especially in studies where neural activity during action production is not measured.

## The Agent

Infants understand object-directed events to be attributes of the actor (the ‘agent’); goals are not generalized from one individual to another (Buresh and Woodward, 2007; Henderson and Woodward, 2012). In this section, we present findings that suggest that the agent who performs the object-directed action provides a number of signals to the observing infant, including their animacy, their history of actions, and their broader bodily movements. In section “The Object Approach,” we will discuss the signals presented by the more fine-detailed mechanics of the agent’s goal-directed action.

### Animacy

Infants appear to limit goal attributions to animate and animated entities, suggesting that distinguishing the animate from inanimate is an important component of this ability. The exact properties of entities that result in the percept of animacy for adults and infants are the topic of a rich body of research (for a review, see Rutherford and Kuhlmeier, 2013) with foundations in the work of Heider and Simmel (1944) and Bassili (1976). Here, we emphasize research that specifically pertains to infants’ recognition of action that is directed to goal objects during the first 2 years of life.

Early, seminal research alluded to the special status of animate (in this case, human) motion to infant goal attribution; in Woodward (1998), infants increased visual attention to a rigid stick moving in a new path of motion rather than to a new goal object, and 18-month-olds in Meltzoff (1995) re-enacted the goal-directed behavior of a human actor but not a machine. Subsequent work has demonstrated that infants encode the actions of agents as directed to particular goal objects if there is evidence that the agent is self-propelled or, relatedly, can change direction (Luo and Baillargeon, 2005; Luo and Johnson, 2009), that the agent can interact contingently with other agents (Shimizu and Johnson, 2004; Johnson et al., 2007), and that the agent is capable of biological motion (Yoon and Johnson, 2009). In these studies, the goal-directed actions are limited to approaching or gazing at objects, owing to the limited physical affordances of the animated agents. As will be seen in section “Hand Conformation,” however, infants appear to consider more fine-grained physical affordances when agents with more articulated appendages (e.g., hands) are depicted.

### History of Goal-Directed Actions

Many studies of infant goal attribution use a procedure in which the infant observes an agent repeatedly performing a goal directed action before an alteration is made to the scene, at which point the infant’s recovery of interest in the scene is measured by looking time. The amount of exposure differs between studies: some use infant-determined habituation paradigms (e.g., those that closely follow Woodward, 1998) while others use familiarization paradigms in which exposure is predetermined by the experimenter (e.g., Luo and Baillargeon, 2005; Hernik and Southgate, 2012). Yet, the fact remains that in these studies, one of the primary pieces of information available to infants regarding what the agent will do in test trials is what the agent has done in the past. Indeed, in more recent studies measuring eye-gaze during the observation of action, infants predict the target of an action, but must see the completed action at least once before doing so (Henrichs et al., 2012; Brandone et al., 2014).

Infants also appear to consider an actor’s new goal directed action in relation to previous action in a different setting. By at least 9 months, infants discriminate between approach behavior to two different characters based on the agent’s previous interactions with the characters in another environment (i.e., helping or hindering, Kuhlmeier et al., 2003; Hamlin et al., 2007; Kuhlmeier, 2013). Relatedly, at the same age, infants who have observed an agent repeatedly manipulate an object in a certain manner (e.g., slide it) look longer if she selects an object that, due to a change in the physical setting, cannot be manipulated in the same way (Song and Baillargeon, 2007). These results suggest that infants are considering not only what an agent is doing in the present, but also what an agent has done in the past. Future studies might consider how others’ past inconsistencies influence infants’ later expectations and predictions.

### Broader Bodily Movements

The agent’s bodily motion, beyond the movement of a reaching arm and hand (see The Object Approach), also appears to be an informative signal relevant to infant goal attribution. Head direction and eye gaze, like a reach, appears to be construed as object directed. Four-month old infants who saw an actor gaze at one of two objects reliably looked less at the object that had been the target of the actor’s gaze, even in the absence of the actor, suggesting that the infants found the object that had not been cued by the actor’s gaze to be more novel (Reid and Striano, 2005). More relevant to the topic of this paper, by at least 12 months of age, infants who were habituated to an event in which an agent gazes toward and smiled at one of two objects later looked longer at events in which the agent held the object that they had *not* previously gazed toward (Phillips et al., 2002). Thus, eye-gaze appears to be interpreted as object directed and may provide information to infants as to an agent’s subsequent object-directed reaches.

Further examples of infants’ use of movement in their interpretation of goal directed action come from infant-directed action, the ‘motionese’ described by Brand et al. (2002). In this study, mothers were asked to demonstrate the properties of five novel objects to either an adult partner or to their own infants. Compared to their interactions with the adults, the mothers’ demonstrations to their infants occurred in closer proximity to the infants, with greater enthusiasm and repetition, and exaggerated but simpler movements. This finding has been expanded upon by others who have demonstrated that parents’ engagement in ‘motionese’ in object-directed actions witnessed by their infants both increases the attention paid by infants to the action and influences the infant’s own later exploration of that object (Brand and Shallcross, 2008; Koterba and Iverson, 2009). Thus, it is possible that outside of laboratory examples of simple object directed reaches, in which ‘motionese’ is typically limited, infants may regularly use these movements in their interpretation of others’ object directed actions.

### Summary and Outstanding Questions

The agent performing an action represents a particularly rich source of information to infants. Infants are capable of interpreting information from the agent’s gaze, from their history with that agent, and from cues that the agent may be providing specifically in an attempt to enhance communication. However, a number of questions remain about what infants take away from this type of information. For instance, while we know that infants use historical information about agents to shape their expectations for those agents, it is unclear exactly how long lasting this influence is, or how durable to changes in the broader environment. Sommerville and Crane (2009), for example, found that 10-month-old infants’ goal attributions may not extend across a change in the room in which the action is occurring. In most goal attribution studies, repeated action is followed closely in time by a test, but it is unclear how readily this translates to infant’s viewing of everyday action.

## The Object Approach

The way that a goal object is approached is also a source of information for infants attending to the action. Indeed, these actions are typically as direct as possible while also being ‘honest’ in that in order to act upon an object, one must necessarily bring themselves into contact with that object in a manner that affords the particular action.

### Direction of Movement

A feature of goal attribution studies that is not often discussed is that the *completion* of the object directed goal is typically witnessed by the infant viewing the reaching. It is thus interesting to consider how infants respond when a portion of their viewing of an action is disrupted. Daum et al. (2008) presented 6- and 9-month-old infants with a video of an experimenter beginning a reach toward one of two objects on a stage, from both the point of view of the experimenter and that of an onlooker. When the experimenter’s hand passed between the midpoint between their starting position and the target object, the video stopped and the infants were simultaneously presented with still frames of completed reaches: a plausible outcome depicting the experimenter holding the object that was on reach trajectory, and an implausible outcome in which the other object was grasped. Infants looked longer toward the displays presenting the implausible outcomes, suggesting that they had formed an expectation as to the target of the reach from the direction of the arm during the initiation of the reach.

Repeated actions that approach the same object but from different starting locations also appear to indicate to infants that, in general, the actions are goal-directed. Evidence for the influence of this ‘equifinal variation’ comes from studies in which infants observe an agent who does not grasp an object, but approaches it through variable routes. In a variation on the Woodward (1998) design, for example, Biro and Leslie (2007) found that 6-, 9-, and 12-month-old infants looked longer when a new object was approached after previously observing a hand or paper tube repeatedly poke a different object from multiple directions. In later studies, both 3-month-old (Luo, 2011) and 6.5-month-old infants (Csibra, 2008) appeared to view the actions of an unfamiliar, non-human agent as directed to a goal object if the agent approached the target object efficiently and with variation in target approach.

### Hand Conformation

At some point during the first year of life, infants begin to consider the appropriateness of the conformation of an agent’s hand to the action they are taking. For example, 9-month-olds who observed repeated, non-functional but object-directed action (an approach with the back of a hand) did not respond to changes in the target object as they do with grasping actions (Woodward, 1999). Similar results have been found in other studies, in which infants made anticipatory gazes toward the target of a grasping reach, but not toward the target of a back-of-hand reach (Kanakogi and Itakura, 2011; Krogh-Jespersen and Woodward, 2014). Additionally, infants are less likely to choose the same target as an experimenter when that experimenter has used a back of hand action (Hamlin et al., 2008). Neuroscience techniques have found converging results. Using EEG, Southgate et al. (2010) found that 9-month-old infants did *not* show motor activation in response to the viewing of a back of the hand action.

Infants also use hand conformation to form expectations about the action that will be performed. Six-month-old infants who were able to produce a pincer grip looked longer when a pincer grasp or a palmar grasp were used on apertures inappropriately sized for those grasps, while infants lacking a pincer grip showed no expectations (Daum et al., 2011). Relatedly, Gredebäck et al. (2009) performed a study in which infants viewed an experimenter either reach for objects and move them across a stage or move a closed fist to each object and then the opposite side of the stage, mimicking the arm movement in the other condition. At 14-months, but not 10-months, infants made predictive gazes to the targets of reaches, but their gaze followed the closed fist reactively. Similarly, as noted in section “Self-Production of the Observed Action,” the pre-shaping of the hand to the size of the target plays a significant role in infants’ ability to predict the target of a reach, depending on their ability to produce that grip themselves (Ambrosini et al., 2013).

### Efficiency to Environmental Constraints

An additional cue comes in the form of the path taken by the agent toward their goal object. This information has a prominent role in one of the most cited models of infant goal attribution (‘the teleological stance,’ Gergely et al., 1995; Csibra and Gergely, 2007). The model posits that the end state of an action may (or may not be) seen as the goal of the action depending on whether the action culminating in that end state is deemed to be efficient in relation to the current environmental constraints. To use a concrete example, by at least 9-months of age, infants consider the goal of an animated ball to be ‘to approach the other ball’ when its means (jumping over a barrier) of getting to this end state is rational given the situational constraints (a barrier is between the balls; Gergely et al., 1995). Similarly, Southgate et al. (2008) found that 6- to 8-month-old infants looked longer at a less efficient motion path (e.g., unnecessarily moving other objects before reaching for a goal object) than a biomechanically impossible motion path (e.g., ‘snaking’ around an obstructing object before reaching for a goal object), suggesting that these infants had a stronger expectation for efficiency of action than for possibility of action. Subsequent research using procedures based on Woodward (1998) and eye-tracking of predictive gaze have further suggested that inefficient action may actually prevent 7- to 12-month-old infants from encoding an action as goal-directed (e.g., Biro et al., 2011; Hernik and Southgate, 2012; Verschoor and Biro, 2012; Biro, 2013).

The empirical focus on the role of action efficiency in infant goal attribution has also led to a critical reappraisal of how we measure whether an infant construes an action as being goal directed. Take, for example, infants’ observation of a reach for an object sitting alone on a table (or in the case of computer-animated agents, an approach toward an object). After habituation or familiarization to this event, infants do not discriminate between reaches for this same object and reaches for a new object that has been added to the table. This null result has been found across many studies and laboratories (Luo and Baillargeon, 2005; Biro et al., 2011; Luo, 2011; Hernik and Southgate, 2012). Some have interpreted these results to suggest that infants do not see reaches toward singly presented objects as being goal-directed because there is no evidence for efficiency of action. Indeed, in conditions in which an agent efficiently circumvents a barrier to get to the object, infants then appear to discriminate between the agent’s actions on the old object versus a newly presented object (e.g., Hernik and Southgate, 2012). Yet, as noted by Kuhlmeier and Robson (2012), it is hard to consider a simple reach toward a single object in the absence of obstacles as anything other than goal-directed and efficient. While it has not been definitively demonstrated that the mirror neuron system is a mechanism for goal attribution in humans, it should be noted that mirror neurons were initially discovered in rhesus macaques because of activation in response to a simple, unimpeded reach toward a single object (di Pellegrino et al., 1992). Indeed, current work on human infants also suggests motor activation in response to the viewing of simple reaches (Nyström, 2008; Southgate et al., 2009).

Instead, it is possible that efficiency of action is particularly important on tasks in which infants must encode the features of a goal object, as is required on tasks in which test trials examine a looking time difference between reaches that continue to be directed to a previous goal object and reaches to a new object (e.g., similar to Woodward, 1998). In these tasks, infants only ‘pass’ if they have initially encoded the agent’s reach as being directed toward ‘that object’ as opposed to being directed toward ‘an object’ (Kuhlmeier and Robson, 2012). Thus, it may be too early to claim that infants do not attribute goal-directedness to agents who reach for singly presented objects, though exactly how the goal object itself is encoded may be limited (see also Object Properties and Presence of Alternatives).

### Summary and Outstanding Questions

From the work reviewed in this section, it is clear that the movement features of the reaching action are a rich source of information to infants. How the action occurs with respect to environmental constraints is a key component to infants’ attribution of goals to others. Biomechanical information, in terms of hand shape and direction of movement, also plays a strong role, particularly in the anticipation of action outcomes. However, a number of questions remain about the processing of this information. In particular, it is worth considering the difference between the functionality of an action and the intentionality of an action. The extension of an arm to place the back of one’s hand on an object is clearly an intentional act, but this action is also treated differently than other actions with a more obvious functionality vis-à-vis an object, on both a neural and behavioral level. Yet, it remains unclear whether infants are failing to attribute a goal or whether the goal of the action seen as something other than the object upon which the action is terminated.

## The Goal Object

One distinct way in which the object acted upon influences the way infants process that action as goal directed is through its very presence or absence. This factor has been especially studied in the mirror neuron literature. Rizzolatti and Craighero (2004), in a discussion of the basic properties of mirror neurons, write (emphasis added):

“There are two classes of visuomotor neurons in monkey area F5: canonical neurons, which respond to the presentation of an object, and mirror neurons, which respond when the monkey sees object-directed action (Rizzolatti and Luppino, 2001). *In order to be triggered by visual stimuli, mirror neurons require an interaction between a biological effector (hand or mouth) and an object*. The sight of an object alone, of an agent mimicking an action, or of an individual making intransitive (non-object-directed) gestures are all ineffective.”

In both adult humans and macaque monkeys, mirror activation has been shown in response to an object-directed action, but not to viewing the same motion performed in the absence of an object (di Pellegrino et al., 1992; Gallese et al., 1996; Umiltà et al., 2001; Muthukumaraswamy et al., 2004). Mirror response in the absence of an object-directed action has been observed, but only in a small minority of studies (Calvo-Merino et al., 2006).

Infants show motor activation in response to an apparently object-directed action but not in response to mimed grasping actions (Southgate et al., 2010). In this experiment, 9-month-old infants were measured with EEG as they watched a demonstrator either make a reaching grasp or a non-functional back-of-hand movement, either in the absence of an object or behind an occluder. Infants exhibited greater motor activation only while observing reaches behind an occluder. Even though the infants could not see the object upon which the reaching object terminated (in this case, one was not actually present), this was the only condition in which the infants could infer an outcome with which they have any experience. Indeed, this is not the only sense in which the availability of the objects matters to infants. For instance, Scott and Baillargeon (2013) demonstrated that infants also consider the mental and physical ease with which objects can be obtained.

### Object Properties

Though the presence of an object appears to be an important factor in infants’ construal of reaching events, by 12-months of age, infants may not differentiate between reaches to objects that are visually different but of the same kind. Following a procedure based on Woodward (1998), Spaepen and Spelke (2007) found that infants who were habituated to reaches toward one of two objects (e.g., a doll) dishabituated to reaches to a new object if the new object was of a different type (e.g., a truck) but not to reaches to a featurally distinct object of the same kind (e.g., another doll). Follow-up experiments revealed that infants could discriminate between the novel objects and the old, but that when the two objects available during habituation were of the same category, the infants did not look longer toward inconsistent choices within that pair at test.

From the first few months of life, object properties have been shown to have an influence on infant looking behavior, in the absence of any goal directed action, and in different ways than seen in adults (Adams, 1987; Henrichs et al., 2012; Taylor et al., 2013). Infants’ own experience with certain object properties, such as weight, also influences the way infants view objects and the actions of others upon those objects. For instance, 11-month-old infants show preferential reaching toward objects they expect to be lighter (Paulus and Hauf, 2011). Infants also show differential *mu* desynchronization when watching an experimenter lift an object based on their experience with that object weight (Marshall et al., 2013a,b).

The affordances of an object, and the infant’s experience with the use of a particular object, also influence infants’ expectations for action on that object For instance, one study found that 12-month-old infants, after watching an experimenter reach for and grasp one of two objects, demonstrated stronger motor cortex activation in response to ‘extraordinary’ events (e.g., phone to mouth, cup to ear) than to ‘ordinary events’ (phone to ear, cup to mouth; Stapel et al., 2010). This result was interpreted as demonstrating increased goal-related planning from the infants as they re-evaluated the unusual action during its execution. In a similar paradigm, Hunnius and Bekkering (2010) examined the anticipatory gaze of infants at a number of ages (6-, 8-, 12-, 14-, and 16-months) and found that infants were more likely to make anticipatory gazes toward the functional target than the non-functional. In another study, it was determined that infants 20-month-old infants, but not 14-month-olds, can predict the intended use of a multi-purpose tool based on the way it is grasped initially (Paulus et al., 2011).

Taken together, these studies suggest certain object properties are more likely to attract infant gaze, influence infants own predilections toward acting upon those objects, and influence the expectations infants have for others’ actions upon those objects. Moreover, infants bring their own experiences with objects to the experiment with them, which can impact the expectations infants have for the actions taken upon those objects. The way that experience with objects influences infants’ expectations for others actions upon those objects is ripe for study (e.g., whether the onset of eating solid food narrows expectations for the types of things that an experimenter might bring to their mouth).

### Occlusion

Though infants are able to process the features of goal objects while they are within view, they have difficulty binding these features to their representation of the object while it is out of view. Yet, a key component of the goal attribution studies discussed thus far is that the objects are typically out of the infant’s sight for some amount of time during a testing session (e.g., when a curtain is lowered or when the locations of the objects are switched^2^). Thus, in order for an infant to show looking time differences for object-directed reaches in a Woodward (1998) style design, the infants must not only have attributed a goal to the actor, but also remember the identity of at least one of the objects that were available to her, even through occlusion. The following section will outline studies demonstrating infants’ difficulty maintaining feature-rich object representations in a way that is robust to occlusion, followed by a discussion as to how this research pertains to infant goal attribution studies.

Experiments on infant object understanding often relate to two concepts, object individuation and object identification. Object individuation refers to the formation of distinct representations for the object/s in question (“there are two things”), while object identification refers to individuating objects and binding at least some of the features of that object to ones representation of that object (“there is a yellow ball and a pink bear”). Research suggests that infants first gain the ability to individuate objects. These spatiotemporally defined representations allow the infant to perform operations such as addition (e.g., 1 object + 1 object = 2 objects: Wynn, 1992), though the identity of the objects is not represented (e.g., 1 Elmo + 1 Elmo = 2 Ernies: Simon et al., 1995).

The earliest ages at which infants have been shown to notice a change in the identity during the presentation of multiple occluded objects is 6-months, at which point infants can identify a single object from a pair, and only if the spatiotemporal distinction is maintained between the objects during occlusion through the use of separate occluders for each object (Kaldy and Leslie, 2005; Kibbe and Leslie, 2011). However, even by 12-months of age, infants still have some difficulty maintaining robust, feature-rich representations of objects that have gone out of sight. For example, after observing a rubber duck and a toy truck emerge one at a time from opposite sides of an occluder, 12-month-olds (but not 10-month-olds) looked longer when the occluder dropped to reveal only one object (e.g., the duck). That is, prior to 12 months of age, infants do not appear to represent the occluded duck that they just saw as a ‘duck’ but as a featureless object that can emerge again as a truck (Xu and Carey, 1996). In a later experiment, the objects used varied either on size, color, and pattern, with the aim of determining which perceptual information would be sufficient to prompt infants to individuate multiple objects in the absence of clear spatiotemporal cues. Here, though, 12-month-old infants did not individuate, succeeding only when the objects differed in kind (Xu et al., 2004).

Findings such as these are difficult to reconcile with the results of goal attribution studies, in which 5- and 6-month old infants (and in some cases, 3-month olds), appear to notice when two objects have switched location while occluded by a single occluder. This situation lacks any spatiotemporal evidence of a change to the objects, and so infants must have bound some features to their representations of at least one of these objects in order to show differential looking time toward an actor reaching their prior goal object versus a different one. It is possible that other factors, of the sorts so far discussed in this review, may be prompting infants in goal attribution studies to form more robust, feature-bound object representations than infants in tasks specifically measuring object identification. This proposal will be addressed again in section “Presence of Alternatives.”

### Action Effects

The binding together of an action and the perceived effect of that action on the world appears to occur from as early as 2-months of age (Rochat and Striano, 1999; Verschoor et al., 2010). In a series of experiments, Verschoor et al. (2010, 2013) demonstrated that infants (ages 7-, 9-, 12-, and 18-months) can bind an action and its effect in a bi-directional way, and that infants after 12-months of age are influenced in their action selection by action effects. Detecting relationships between actions and their effects also impacts infants’ understanding of others’ object directed reaches. In previous sections, we noted that when an infant views non-functional back-of-hand action toward an object, they show different neural response than to grasping actions and also do not show changes in looking time when the target of the action is changed (Woodward, 1999; Southgate et al., 2010). However, when this back-of-hand action is presented along with a salient action effect, such as moving the object contacted, infants become sensitive to later changes in the target of the action (Király et al., 2003; Biro et al., 2014).

Further evidence for binding between object directed actions and their effects comes from measures of *mu*-desynchronization. Paulus et al. (2012) demonstrated that 8-month-old infants show greater *mu*-desynchronization when presented with the sound of a special rattle they had been trained to use, compared to other familiar and unfamiliar sounds. Nine-month-olds, when presented with the sounds from a rattle that they have only ever seen shaken, show increased motor activation, despite having never produced the action to cause that sound before themselves. In sum, infants appear to bind novel action effects to their motor representations of actions already within their motor repertoire (Paulus et al., 2013).

### Summary and Outstanding Questions

In this section, we have presented aspects of the goal object that appear to be relevant to infants’ understanding of object-directed action. The mere presence or absence of an object and whether or not the action upon the object has any perceptible effect have a strong influence both on the neural processing of the object-directed action and, relatedly, upon the infants behavior in response to the action. The features of the targeted object and its affordances also influence infants’ expectations for others actions upon them.

Of particular interest is how infants’ ability to represent objects during occlusion is influenced by goal-directed action upon those actions. As brief occlusion of objects has been a commonplace feature of goal attribution studies since Woodward’s (1998) original study, the discrepancy between the ages at which infants successfully encode the features objects in goal attribution studies (5–6-months, 3-months under certain circumstances) and object cognition studies (12-months, 6-months under specific circumstances) is worthy of examination.

## The Goal Environment

### Presence of Alternatives

As noted in section “Efficiency to Environmental Constraints,” after observing an actor reach for an object sitting *alone* on a table, infants up to at least 9 months of age do not subsequently discriminate between reaches to this same object and reaches for a new object that has been added to the table (e.g., Biro et al., 2011). By some accounts, infants’ difficulty in this task is due to the fact that though infants can attribute to agents both goals and preferences for objects, in the case of a reach or approach behavior directed to a lone object, there is no evidence regarding the agent’s preference between the original object and the newly added object. Thus, in contrast with infants tested with the Woodward (1998) paradigm, infants have no basis for distinguishing between subsequent reaches to the old object or to the new one (e.g., Luo and Baillargeon, 2005). By another account, infants do not distinguish between reaches to the old and new object because they never encoded the original reach toward the single object as being goal-directed in the first place (Hernik and Southgate, 2012).

A third possibility, though, is that that infants fail to demonstrate expectations for action in a single-object condition because they did not encode the *specific* object the agent interacted with, not because they failed to perceive the action toward the single object as goal-directed or had no information regarding the actor’s preferences. As noted earlier, tasks that are based on Woodward (1998) require infants to encode the features of objects that are reached for, and it is possible that the presence of an alternative object might influence infants’ processing of the object that is being acted upon. In support of this claim, 9-month-old infants do not rely on the *identity* of a secondary, unchosen object as a prompt to encode the feature of an actor’s target object, but do seem to rely on its mere presence (Robson and Kuhlmeier, 2013).

Yet, in other situations, the identity of potential alternative goal objects may be informative for infants in interpreting the object-directed reaches of others. For example, in one study, 9-month-old infants were tested in a paradigm involving multiple object pairings (Robson et al., 2014). Two objects (A and B) were present on a stage in front of an actor, who would reach out and choose one of these (A). In the next trial, there were also two objects on stage; this time the pair contained one object that had been seen before and one new object (B and C). Now, the actor reached for the previously ignored object (B). These trials alternated until infants reached habituation, at which point infants were shown just one of the pairings (A and B or B and C) and shown reaches that were either consistent or inconsistent (e.g., B when A and B were present) with the actor’s previous goals. Infants looked longer toward inconsistent actions, suggesting that they must have been encoding not only the features of the target objects, but also of the alternatives. That is, a reach for B was inconsistent in the presence of A, but not in the presence of C. Using a similar method, 16-month-old infants were shown to demonstrate transitive inference, which again would rely on encoding the identity of the alternative objects (Mou et al., 2014). Thus, it is possible that infants may encode the features of alternatives when doing so is necessary to form expectations about which actions another might take.

### Location of Action

Sommerville and Crane (2009) investigated 10-month-old infants’ ability to represent goals across locations. They provided infants with unambiguous information about an actor’s choice of objects by having the actor select one of two objects from a spot on the floor prior to testing. The infants then habituated to the selection of one object from a pair through means infants typically find ambiguous, either in the same room in which the pre-test reaching occurred, or in a different room. It was found that infants interpreted the ambiguous action as being about the target object only when the test was performed in the same room as the pre-test reaches toward that object. This suggests that, at 10-months of age, infants’ representations of others goals may not be durable to changes in the broader setting.

However, in other studies, changes of scenery have not disrupted infants’ representations of others’ goals. As noted in section “History of Goal-Directed Actions,” by 9 months, infants discriminate between an agent’s approaches to two different characters based on that agent’s previous interactions with the characters in another environment (i.e., Kuhlmeier et al., 2003; Hamlin et al., 2007). At the same age, infants who have observed an agent repeatedly move an object in a certain manner look longer if she selects an object that, due to a change in the physical setting, cannot be moved in the same way (Song and Baillargeon, 2007). A potential explanation for this discrepancy could be that in experiments where infants attribute goals across settings, it is the agent’s location that has changed, while the infant has remained in the same place. However, further study of the durability of infants’ attribution of goals to changes in setting is necessary for making stronger claims about why this occurs in some cases and not others.

## Conclusion

In this review we have discussed how context can influence the way an infant processes the object-directed actions of others. We have drawn from research on the mirror neuron system, object understanding, and infant goal understanding, compared findings and methodologies across these disciplines, and discussed how findings from each of these domains may have implications for the others. The primary goals of this review were to provide a relatively broad, though likely not exhaustive, review of several research areas and discuss their relevance to the problem of understanding infant goal attribution. The secondary aim was to highlight the variety of sources from which infants can draw information to inform their expectations of others’ object-directed actions. In doing so, we have presented a number of observations and open questions pertaining to each of these information sources, but in concluding will attempt to do so on a broader scale.

One of the most readily apparent directions forward is to study how these various sources of information available to infants work together, or how infants prioritize these sources when they conflict. As a result of our striving to perform controlled experiments, we often create artificial environments in which only one of these many factors is variable, when in fact any of them could potentially matter and almost all of them will be variable in the real life situations in which infants actually employ goal attribution as a mechanism for learning. Broadly speaking, motor system activation, personal experience and physical ability, and teleological considerations all appear to contribute greatly toward infants’ understanding of the goals of others, with none of these appearing to be able to explain this ability completely in isolation. Comparing ages and achievements across object cognition and goal attribution literature seems to point to concurrent changes in these cognitive abilities at around 5–6 months of age that make goal attribution possible. Further evidence would be required to make strong claims regarding simultaneous changes in the mirror neuron system at 5–6 months. However, as infants are developing their own reaching capabilities at this age, and given the change in infant behavior toward others reaches as their own grasping competence grows (Gredebäck et al., 2009; Daum et al., 2011; Ambrosini et al., 2013) and the evidence for change in mirror neuron function in response to experience (Ferrari et al., 2005; Umiltà et al., 2008; Rochat et al., 2010), such changes seem plausible.

Attributing a goal to someone else is complicated, and there is a great deal of information available to an infant watching an object-directed action that can influence their interpretation of that goal. It is important consider all of these factors, not only to form a fuller understanding of this phenomenon, but to inform our thinking about how the methods we use can constrain how we conceptualize an ability.

## Author Contributions

Both authors made substantial contributions to the conception, writing and editing of this work. Both authors have approved this work for publication and agree to be accountable for all aspects of the work.

## Conflict of Interest Statement

The authors declare that the research was conducted in the absence of any commercial or financial relationships that could be construed as a potential conflict of interest.

## Footnotes

1 In most cases in this review, an object is a discrete and visually distinct artifact of a size easily graspable by an experimenter, such as a teddy bear or a ball, as this is typical of what is presented in the majority of the experiments discussed. However, it is useful to recognize that the category ‘object’ is exceptionally broad and could potentially encompass nearly any physical feature of the infant’s environment, including the bodies of agents or that of the infant themselves.

2 A notable exception to this occlusion during the changing of the location of test objects (the only example of which the authors are aware) is found in Spaepen and Spelke (2007). In this work, two experiments occurred in which infants were habituated to a pair of objects of the same category (two distinct trucks or two distinct dolls), and their locations were switched before test within the view of the infants. This was done to examine whether the loss of spatiotemporal information was to blame for infants failure to show looking time differences toward inconsistent action upon objects of the same category when the location switch was occluded. As all three of these experiments presented null results; however, the conclusions that can be drawn from these results pertaining to the current discussion are limited.
